# Gene Expression and Pulmonary Toxicity of Chitosan-*graft*- Polyethylenimine as Aerosol Gene Carrier

**Published:** 2013

**Authors:** Jung-Taek Kwon, Hu-Lin Jiang, Arash Minai-Tehrani, Chang Gyu Woo, Mansoo Choi, Chong-Su Cho, Yeon-Soo Kim, Myung-Haing Cho

**Affiliations:** a *Laboratory of Toxicology, College of Veterinary Medicine, Seoul 151-742, Korea.*; b *Nanotechnology and Thermal Processing Laboratory, School of Mechanical and Aerospace Engineering, Seoul 151-742, Korea*; c *Research Institute for Agriculture and Life Sciences, Seoul National University, Seoul 151-742, Korea. *; d *Indang Institute of Molecular Biology, Inje University, Seoul 100-032, Korea.*; e *Department of Nanofusion Technology, Graduate School of Convergence Science and Technology, Seoul National University, Suwon 443-270, Korea.*; f *Advanced Institute of Convergence Technology, Seoul National University, Suwon 443-270, Korea.*; g *School of Pharmacy, China Pharmaceutical University, Nanjing 210009, China. *

**Keywords:** Aerosol delivery, Chitosan, Gene carrier, Polyethylenimine, Pulmonary toxicity

## Abstract

Chitosan-*graft*-polyethylenimine (CHI-*g*-PEI) copolymer has been used for the improvement of low transfection efficiency of chitosan. The present study aims to test the pulmonary toxicity and efficiency of CHI-*g*-PEI as an aerosol gene carrier. Mice were exposed to aerosol containing green-fluorescent protein (GFP)-polyethylenimine (PEI) or GFP-CHI-*g*-PEI complexes for 30 min during the development of our nose-only exposure chamber (NOEC) system. CHI-*g*-PEI-mediated aerosol delivery demonstrated 15.65% enhancement of the fluorescence intensity. Compared to PEI, CHI-*g*-PEI showed no significant pulmonary toxicity. In summary, using CHI-*g*-PEI is safe and shows high transfection in aerosol gene delivery to animals, and enhanced efficiency was achieved through our aerosol gene delivery system. Therefore, CHI-*g*-PEI and this system would be applicable to future study for aerosol gene therapy.

## Introduction

Aerosol delivery of therapeutic gene is made directly to the respiratory tract without first delivery to other organs and tissues. Due to these advantages, many studies have been applied to gene therapy for lung disease, such as lung cancer (1), asthma (2) and chronic obstructive pulmonary disease (3) using aerosol delivery. Aerosol therapeutic delivery system consists of aerosol generator, delivery-chamber, drug-carrier and air flow controller. 

The gene carriers are broadly classified into two groups: viral and non-viral vector systems. Non-viral gene carriers were attracted increasingly because of their many advantages such as low immunogenicity, large-scale production and safety (4). Chitosan, a natural polysaccharide, is safe, biocompatible and mucoadhesive materials with high cationic charge potential (5). However, this material has low transfection efficiency as a result of weak release from endosomes into the cytoplasm (6).

In our previous study, we have overcome this problem by grafting with the polyethylenimine (PEI) (CHI-*g*-PEI) (7). However, pulmonary toxicity and gene expression by *in-vivo *aerosol gene delivery have not been investigated yet. Therefore, in the current study, we present information from both gene expression and pulmonary toxicity in the lung through our aerosol gene delivery system and CHI-*g*-PEI as an aerosol gene carrier.

## Experimental

*Materials*

Chitosan (molecular weight, 100 kDa; deacetylation degree, 87.7%) was kindly supplied from Jakwang (Ansung, Korea). Branched PEI 25K was obtained from Sigma-Aldrich (St. Louis, MO, USA). Branched PEI 1800 Da was purchased from Wako (Osaka, Japan). pcDNA3.1-GFP was purchased from Invitrogen (Carlsbad, CA, USA). The plasmids were propagated in *E. coli*, extracted by the alkalilysis technique, and purified by QIAGEN kit (Chatsworth, CA, USA).

*Aerosol gene delivery system*

**Figure 1:**
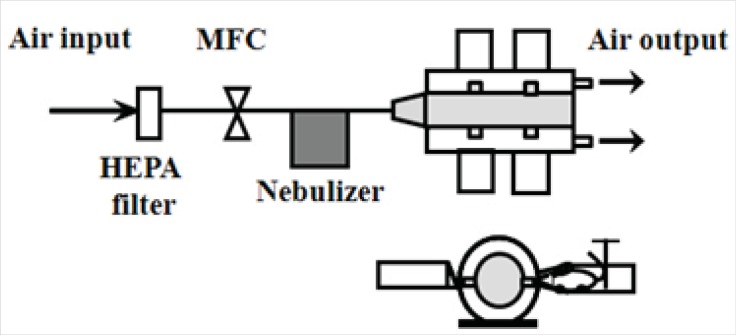
Schematic diagram of aerosol gene delivery devices. HEPA filter: High-Efficiency Particulate Air filter. MFC: Mass Flow Controller.

The nose-only exposure chamber (NOEC) system consists of dual cylindrical box and 4 small tubes (Figure 1). 

Conical acrylic tubes were connected to the main chamber and mice (male C57BL/6, 8~10 wks old, SLC, Hamaguchi, Japan) were placed in the tubes. All animal experiments were performed in accordance with the guidelines of Seoul National University for the care and use of animals. Nebulizer (Dusturbo, Seoul, Korea)-generated aerosols were entered into the NOEC. Operation of the aerosol generation process by nebulizer was as follows: air flow rate was 2 L/min (*lpm*) by a mass flow controller (MFC, Brooks Instruments, Hatfield, PA, USA) and the complex was composed of plasmid DNA; 200 μg and polymer (PEI 25K; 260 μg, N/P ratio of 10 and CHI-g-PEI; 1,400 μg, N/P ratio of 35) was added to 20 mL deionized water. Aerosol size distributions were measured with a dust monitor (Grimm Aerosol Technik, Germany). Size distribution measurements were taken in the NOEC.

*Transfection efficiency*

To determine the efficiency of gene transfection, animals were exposed to aerosol containing GFP-PEI and GFP-Chi-*g*-PEI complex for 30 min in NOEC system. Animals were sacrificed 48 h after the inhalation and routinely fixed lungs were cryosectioned for confocal laser scanning microscopy (CLSM, Carl Zeiss-LSM510, Germany). CLSM images were quantified and analyzed by a computerized system (Media Cyber-netics, Silver Spring, MD, USA).

*Pulmonary toxicity analysis*

For the assessment of pulmonary toxicity of CHI-*g*-PEI and PEI, mice were exposed to aerosols, two times in a week for a total of 4 weeks. The control group was exposed to air filtered by a high-efficiency particulate air (HEPA) filter and polymer groups were exposed to aerosol containing PEI; 260 μg and CHI-g-PEI; 1,400 μg without DNA in distilled water, respectively. At the end of exposure, bronchioalveolar lavage (BAL) fluid from mice was obtained by whole-lung lavage. As a marker of cellular damage, lactate acid dehydrogenase (LDH) activity in BAL fluid was measured using an automated biochemical analyzer (VITALAB, Merck, the Netherlands).

*Histopathological examination*

For histopathological analysis, lungs were removed from each animal. The organs were immersion-fixed in 10% neutral buffered formalin. After routine tissue processing, the tissues were embedded in paraffin and the tissue sections (5 μm) were then prepared for hematoxylin and eosin (H&E) and Periodic acid-Schiff (PAS) stain. The slides were evaluated under light microscopy.

*Statistical analysis*

All results are expressed as mean ± standard error. A multiple variance of a Student’s t-test (Graphpad Software, San Diego, CA, USA) was used to compare the test groups with those obtained from unexposed control group. The level of significance was set at p < 0.05 and p < 0.01.

## Results and Discussion

In this study, we investigated the gene expression and pulmonary toxicity of CHI-*g*-PEI as aerosol gene carrier. We found that CHI-*g*-PEI is safe to use and shows higher transfection than PEI in aerosol gene delivery to animals, and enhanced efficiency was achieved using our aerosol gene delivery system.

*Aerosols characterization*

Geometric Mean Diameter (GMD) of nebulized aerosols in N-OEC measurements was as follows: GFP-PEI; 0.39 and GFP-CHI-*g*-PEI complex; 0.42 μm, respectively. Geometric Standard Deviation (GSD) was 2.46 and 2.26, respectively. Our study demonstrated that GMD results obtained from GFP-PEI were not different from those of GFP-CHI-*g*-PEI. However, the horizontal chamber is easy to handle and has a space saving in the aerosol delivery system. The mean diameters of generated aerosols over 1 μm are known to be increased with solute concentration of the solution (8); however, the solute concentration of generated aerosols under 1 μm size can be different from that of solution in the nebulizer reservoir (9). Measured size distributions showed that large portion of generated droplets are smaller than 1 μm, so the efficiency of trasfections might be different for each case.

*Transfection efficiency*

**Figure 2:**
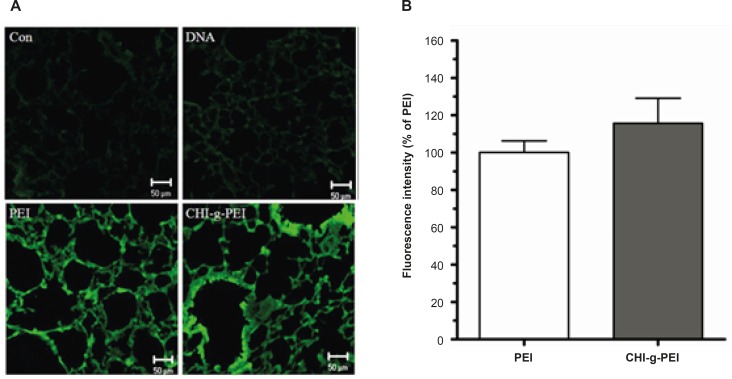
Transfection efficiency in the lung. (a). Analysis of GFP expression in the lungs of mice. Scale bar = 50 μm. (b) Data are expressed as a percentage of the fluorescence intensity as compared with the PEI group. (n = 4, mean ± SE).

As shown in Figure 2(a), compared with the control group, the green intensity of GFP was dominant in the polymer group (PEI and CHI-*g*-PEI). In addition, the CHI-g-PEI group showed 15.65% enhancement of the fluorescence intensity as compared to the PEI group (Figure 2b). It was already reported that polyethylenimine-graft-chitosan (PEI-g-chitosan) showed higher gene expression than PEI (25 K) in liver due to the higher level of amine content in the DNA-carrier complex (10). Furthermore, gene expressions by PEI are affected by the serum (11, 12), whereas, chitosan increased the transfection efficiency with serum (13). The gene expression of low molecular weight polyethylenimine grafted N-maleated chitosan (NMC-g-PEI) was not affected in the presence of serum (12). In a previous study, we also observed higher transfection efficiencies with CHI-*g*-PEI compared to PEI in the presence of serum (7). Taken together, these results indicate that CHI-*g*-PEI with serum in the lung enhances the efficiency of the aerosol gene delivery.

*Pulmonary toxicity*

**Figure 3:**
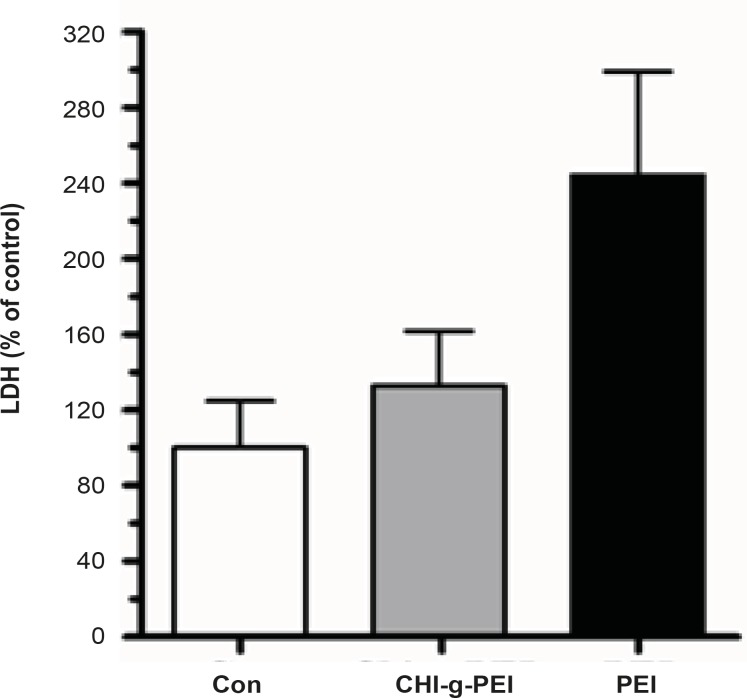
BAL fluid analysis. Comparative pulmonary toxicity of gene carrier**. **Analysis of LDH level in the BAL fluid. (n = 3, mean ± SE).

**Figure 4:**
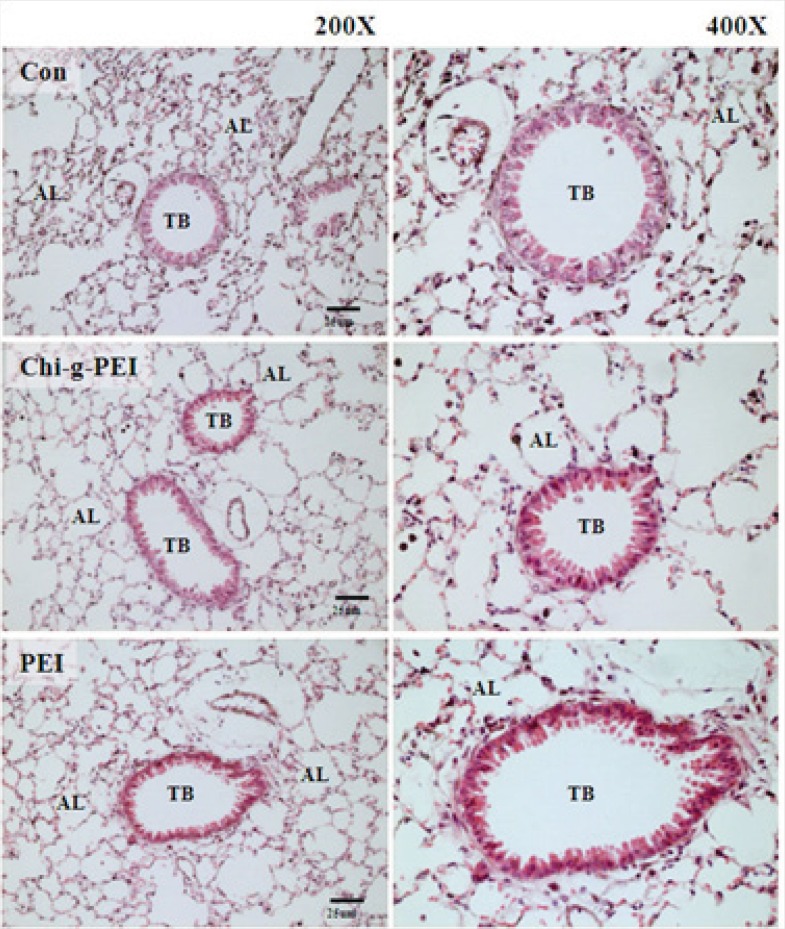
histopathological findings in mice exposed to aerosol gene carrier. Hematoxylin and eosin (H&E) section of the lung. AL: alveoli. TB: terminal bronchial. Scale bar = 25 μm.

**Figure 5:**
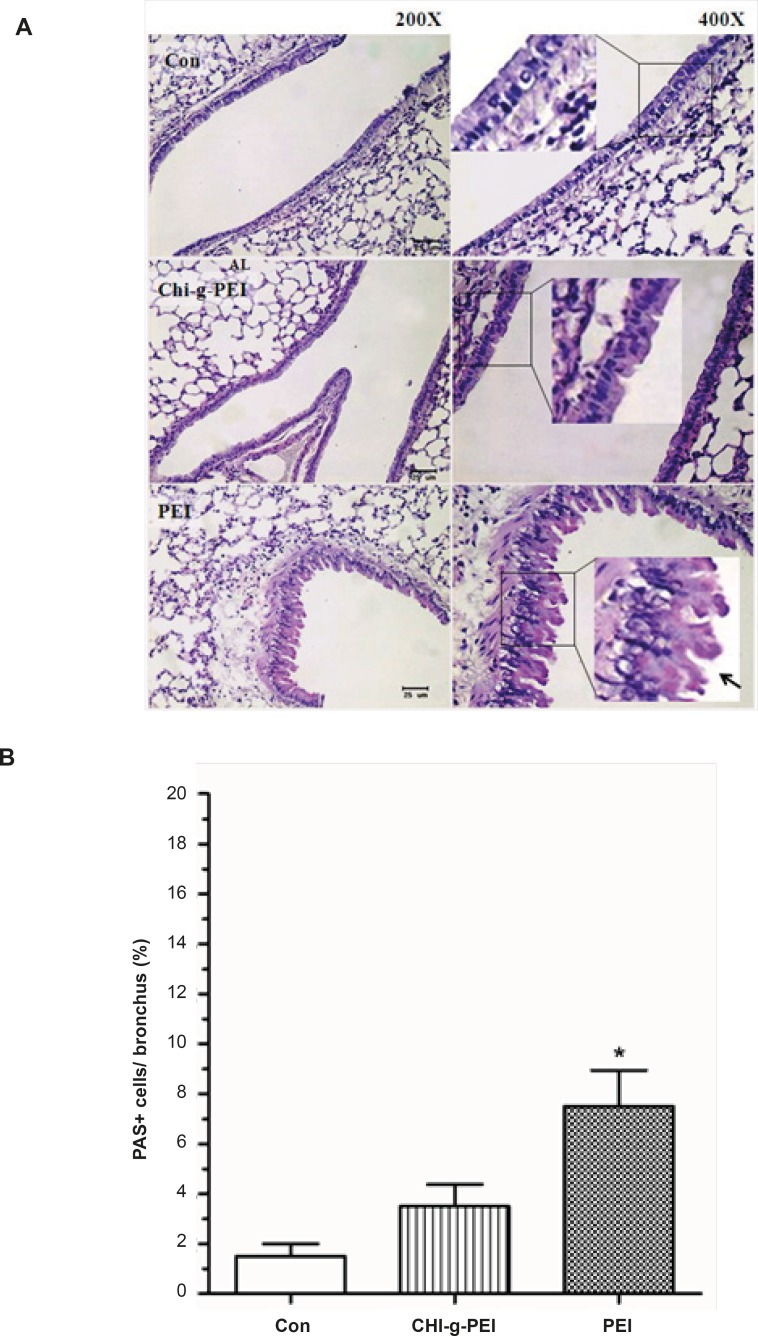
Mucin production of the lungs in mice after polymer exposure. (A) Periodic acid schiff (PAS) stained lung sections. Square box shows goblet cells. Arrow: mucin-producing (bright purple in the lumen). Scale bar = 25 μm. (B) Area of mucin staining percent of airway epithelium. n = 4, mean ± SE. Significant difference with control group, *p < 0.05.

Our results showed that LDH levels in BAL were higher in the PEI group compared to the CHI-*g*-PEI group (Figure 3). H and E analysis of the CHI-*g*-PEI and PEI groups showed no detectable change compared to the control group (Figure 4). However, the results of our present experiment stained PAS showed that CHI-g-PEI was secreted small amount of mucin compared to the PEI (Figure 5). Mucin has high molecular weight and is produced by epithelial tissues and they protect our body from toxicants by forming the mucosal barrier (14). Moreover, mucin secretion indicated that repeated inhalation of PEI could induce cellular immune responses in the lung. Complexes of PEI with mucin break the PEI/DNA interactions and it probably reduces the gene expression in the lung (15).

We showed that PEI has high cytotoxicity in diverse cell line types (7). However, some studies of aerosol delivery to animals using PEI investigated the difficulties with toxicity (16). Apparent discrepancies between our results for PEI toxicity and those of earlier studies may be due to differences in design. In our study, we used the N-OEC system with repeated inhalation of polymer, whereas previous studies applied a whole-body exposure chamber (W-BEC) system with single exposure. W-BEC is frequently used in long-term inhalation studies, as it is suitable for efficient delivery of a large number of animals without restraint. However, W-BEC requires a large amount of test materials; thus, this method limits practical application. On the other hand, our N-OEC has various advantages, including simple control, low cost, and suitability for repeated aerosol delivery. 

In summary, CHI-*g*-PEI was safe to use and showed higher transfection than PEI in aerosol gene delivery to animals, and enhanced efficiency was achieved by use of our aerosol gene delivery system. Therefore, CHI-*g*-PEI has the potential to be safe and efficient gene carrier and this system would be applicable for future studies. 
